# The American Medical Association

**Published:** 1860-07

**Authors:** 


### THE AMERICAN MEDICAL ASSOCIATION.

The Thirteenth Annual Convocation of this body took place on the 5th of June, at New Haven, Connecticut. The well-known attractions of the beautiful City of Elms drew together a large number of the members of the profession. The citizens of New Haven entertained their guests in a most genial and hospitable manner, and made it an occasion long to be remembered with pleasure by all who participated in its enjoyments.

The great majority of the delegates were from New England and New York, the Southern and Northwestern States being but lightly represented.

Dr. Eli Ives, of New Haven, was elected President for the ensuing year; a compliment both to himself and to the city of his residence, eminently merited.

The Vice Presidents are Drs. Wilson Jewell, of Pa.; A. B. Palmer, of Mich.; Joseph P. Logan, of Georgia; John B. McDowell, of Mo.

Treasurer—Dr. Casper Wistar, of Pa.

Secretaries—Drs. S. G. Hubbard, of Ct.; H. A. Johnson, of Ill.

Committee of Arrangements—Drs. N. S. Davis, J. W. Freer, De Laskie Miller, E. Andrews, H. W. Jones, Thomas Bevan, and J. W. Bloodgood, all of Chicago.

On Prize Essays—Drs. Daniel Brainard, Ill.; M. L. Seaton, Mo.; D. S. McGugin, Iowa; John Evans. Ill.; A. G. McArthur, Ill.

On Publication—Drs. F. G. Smith, Pa.; S. G. Hubbard, Ct.; E. D. Hartshorn, Pa.; Caspar Wistar, Pa.; R. J. Breckenridge, Ky.; H. F. Asken, Del.

The Association adjourned to meet in Chicago on the first Tuesday in June, 1861.

The Convention of Medical Teachers met the day before the Association, in accordance with their adjournment at Louisville, Ky. The representation of Medical Schools was not very general, and they did not recommend changes of any importance in the plan of medical instruction heretofore followed.

